# A multidisciplinary study on the social customs of the Tang Empire in the Medieval Ages

**DOI:** 10.1371/journal.pone.0288128

**Published:** 2023-07-26

**Authors:** Dongyue Zhao, Yang Chen, Gaowen Xie, Pengcheng Ma, Yufeng Wen, Fan Zhang, Yafei Wang, Yinqiu Cui, Shizhu Gao

**Affiliations:** 1 School of Cultural Heritage, Northwest University, Xi’an, China; 2 School of Pharmaceutical Sciences, Jilin University, Changchun, China; 3 Xianyang Institute of Cultural Relics and Archaeology, Xianyang, China; 4 School of Life Sciences, Jilin University, Changchun, China; University of Otago, NEW ZEALAND

## Abstract

Multidisciplinary research on human remains can provide important information about population dynamics, culture diffusion, as well as social organization and customs in history. In this study, multidisciplinary analyses were undertaken on a joint burial (M56) in the Shuangzhao cemetery of the Tang Dynasty (618–907 AD), one of the most prosperous dynasties in Chinese history, to shed light on the genetic profile and sociocultural aspects of this dynasty. The archaeological investigation suggested that this burial belonged to the Mid-Tang period and was used by common civilians. The osteological analysis identified the sex, age, and health status of the three individuals excavated from M56, who shared a similar diet inferred from the stable isotopic data. Genomic evidence revealed that these co-buried individuals had no genetic kinship but all belonged to the gene pool of the ancient populations in the Central Plains, represented by Yangshao and Longshan individuals, etc. Multiple lines of evidence, including archaeology, historic records, as well as chemical and genetic analyses, have indicated a very probable familial joint burial of husband and wives. Our study provides insights into the burial customs and social organization of the Tang Dynasty and reconstructs a scenario of civilian life in historic China.

## Introduction

The Tang Dynasty (618–907 AD) was one of the most prosperous dynasties in Chinese history. Its political and economic system, together with social customs, had a great impact on the subsequent Chinese and even wider Asian societies. As the capital of the Tang Dynasty, Chang’an was located in the Central Plains, an area with favorable natural conditions. Sustained development and construction for the Qin to Sui Dynasty meant that by this period Chang’an had become an international metropolis with the highest level of social and cultural prosperity in that period. In the mid-Tang Dynasty, economic development, coupled with the successive implementation of the imperial examination system and new tax laws broke the monopology on political and economic resources that has been held by the aristocracy. Stratification and reorganization of the social classes began to take place. Consequently, common civilians had become the main stratum of society, and played an increasingly important role in economic and political life [1, 2]. However, formal historical records tend to focus only on important figures and events [3]. There are very few detailed records on the lives and customs of ordinary civilians despite there being many official historical materials related to the Tang Dynasty [3].

In recent years, with the continuous integration of multiple types of information from ancient DNA and biogeochemistry to history and archaeological study, multidisciplinary approaches have greatly enriched our knowledge of ancient societies and revitalized historical narratives [4–8]. Genome-wide data enables researchers to infer ancestral elements of populations or individuals in different regions with high precision and geochemical analysis of artifacts and biological tissues has become critical for studying human migration and trade routes [4, 5, 7, 8]. For example, Bongers et al. integrated genomic, archaeological, historical, and biogeochemical data to investigate six individuals from two cemeteries in southern Peru, and got consistent results that supported a Late Horizon population moving from the north Peruvian coast to the Chincha Valley [5]. Furthermore, environmental data [9], stable isotope proxies for palaeo-diet [10], analysis of organic residues [11], as well as many new scientific approaches, such as collagen fingerprinting and palaeoproteomics [12, 13], are used to reveal more comprehensive information of the human history.

**Fig 1:**
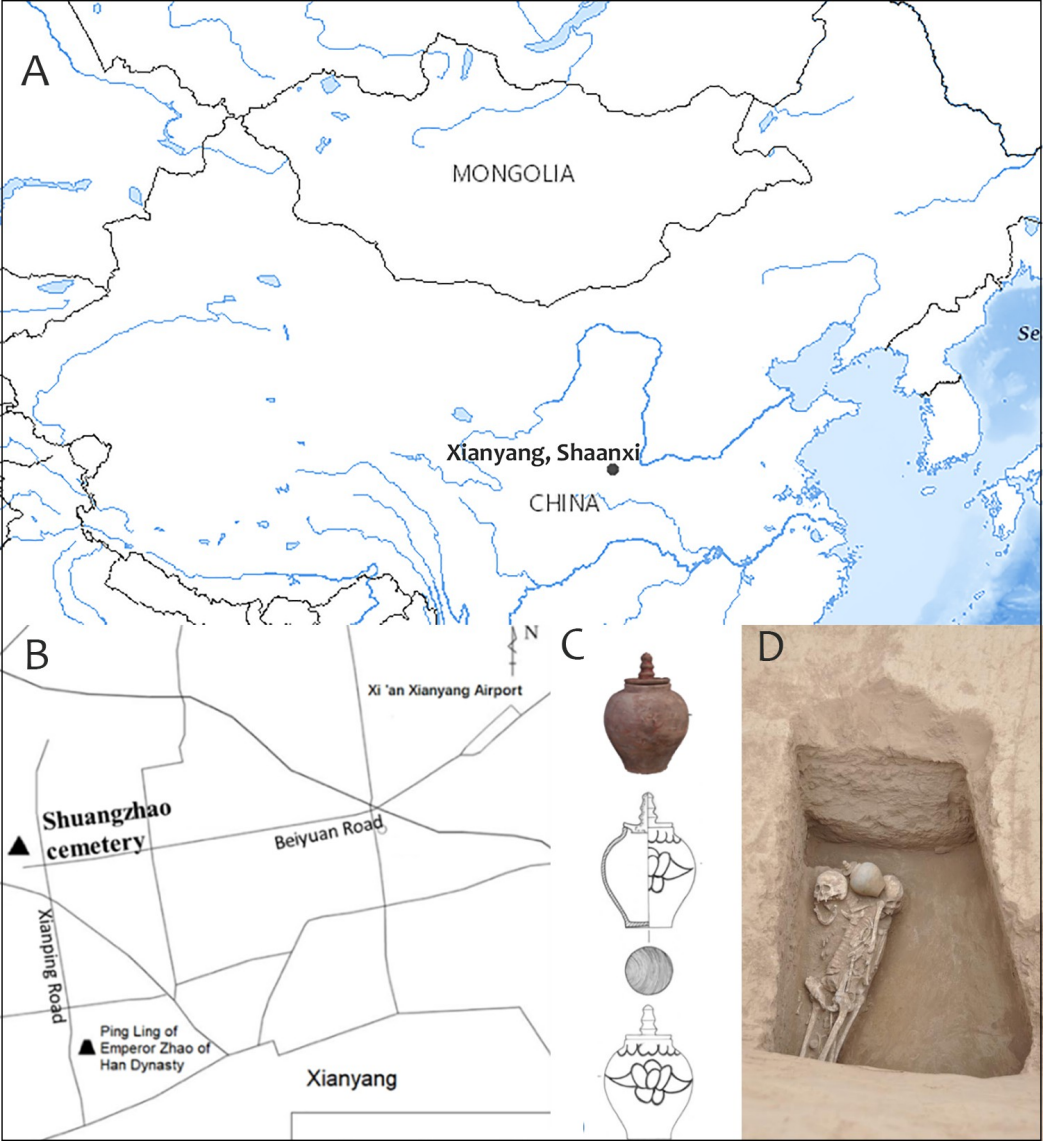
The geographic location of the Shuangzhao cemetery and archaeological excavations of M56. A. The location of Xianyang, based on map from USGS National Map Viewer (public domain): http://viewer.nationalmap.gov/viewer/. B. The diagram of the location of the Shuangzhao cemetery (Map made by authors using Adobe Photoshop). C. The pagoda-shaped jar recovered from M56 (Map made by authors using Adobe Photoshop). D. The photo of M56 taken from the top.

Although there are many historical materials from the Tang Dynasty, such as epitaphs and murals in tombs, which can provide useful information for archaeological study [14, 15], human bone material from the Tang Dynasty is rare. To our knowledge, there has been no published data in the bioarchaeological study of the Tang Dynasty so far, which involves genomic, physical anthropological, and palaeodietary analysis. In 2019, during the excavation by the Xianyang Institute of Cultural Relics and Archaeology in the Shuangzhao cemetery in the Xianyang of Shaanxi (Fig 1A), archaeologists discovered a Tang Dynasty tomb (M56) with well-preserved human remains, providing us with a valuable opportunity to get a first glimpse into the lives of ordinary individuals in the Tang Dynasty. Xianyang, adjacent to the Capital Chang’an, was under the jurisdiction of Jingzhaofu in the Tang Dynasty. A large number of aristocrats and common civilians were buried there [15, 16]. A research team composed of multidisciplinary researchers in the field of archaeology, anthropology, isotope chemistry, molecular biology, conducted a cross-disciplinary investigation on the historic individuals from the Shuangzhao cemetery. We employed archaeological analysis to provide insights into the funeral customs, material culture, chronological sequences and social organization of this cemetery. Osteological analysis was conducted to describe the sex, age, physical characteristics and health status of the buried individuals. We generated carbon and nitrogen stable isotope data to shed light on the diet of the Tang Dynasty people. Genomic evidences permitted us to obtain information on the kinship relations and genetic composition of the Shuangzhao individuals. In this study, we aim to reconstruct the social customs of the civilian class in the Middle Tang Dynasty to the greatest extent, and establish a new method of modern, multi-disciplinary archaeological research.

## Materials and methods

### Archaeological context

The Shuangzhao cemetery is located in Xianyang City, Shaanxi Province, China (Fig 1). This cemetery was excavated by Xianyang Municipal Institute of Cultural Relics and Archaeology in 2019–2020. A total of 59 burials were unearthed, including one burial of the Tang Dynasty (M56), and 58 burials of the Han Dynasty. M56 (cal 1268–1072 BP) in the Shuangzhao cemetery, is 6.76 meters long and consists of a slope tomb passage and a coffin chamber, situated in the northern part of this cemetery. M56 exhibits the features of the middle and late Tang Dynasty (8th century to early 10th century) tombs: a straight-back knife shaped plane structure, with the east wall of the tomb passage and chamber on the same plane [17]. The tomb chamber is 2.06 meters long, 1–1.37 meters wide, 0.9–1.3 meters high. Three sets of human remains (numbered R1, R2 and R3 respectively) and a pagoda-shaped jar with a lid (Fig 1D, S1 Fig in S1 File), a typical artifacts of the Tang Dynasty, were found in the west part of the chamber, with R1 laying on the top of R3 and to the west of R2 (Fig 1C, S1 Fig in S1 File). The access to the human remains was approved by the Xianyang Municipal Institute of Cultural Relics and Archaeology. No permits were required for the described study, which complied with all relevant regulations. The skeleton of R1 remains intact and in the primary anatomical position (S1 Fig in S1 File). R2 was placed a little lower than R1 (S1 Fig in S1 File). Her humeri, hips, femora and ribs were not in the original anatomical position. After the excavation of R1, the skeleton of R3 was exposed. The bones of R3 were scattered (S1 Fig in S1 File). This suggested that R2 and R3 had experienced disturbance or secondary burial. The architectural structure of the tomb showed that M56 remained original construction with no signs of secondary use. Thus, the inhumation of the three individuals in M56 was simultaneous. Considering the skeletal layout of these individuals and the funeral customs of returning the remains in the Tang Dynasty [18–20], we speculated that R1 was deposited in M56 primarily, and R2 and R3 were moved from their original burial to M56.

### Archaeological analysis

As the capital of the Tang Dynasty and the territory of the Jingzhaofu, Xi’an and its surrounding areas were places where lots of common civilians and nobles were buried [15, 16]. By the end of 2008, more than 800 cemeteries of the Sui and Tang Dynasty had been found in Shaanxi [16]. The funerary rituals of the Tang Dynasty are well-understood and archaeologists can examine grave type and artifact assemblages to establish the chronological sequence of burials and identify the social position of the tomb owner. The age of burial M56 is determined by the burial structure and specification, based on the criteria established by the Institute of Archaeology of the Chinese Academy of Sciences [17]. The pagoda-shaped jar buried in M56 is an artifact, which firstly appeared in the Tang Dynasty and continued being used from the early Tang Dynasty to the late Tang Dynasty, with well-regulated structure changing. Therefore, it becomes one of the typical objects to estimate the date of Tang graves in the northern areas [21].

### Osteological analysis

Three adult individuals were found in M56. The remains’ sex was mainly determined by the morphology of the pelvis and cranium [22]. Their ages were generally estimated on the morphology of the pubic symphysis [22], the morphological changes of the auricular surface of the ilium [23], the obliteration of cranial sutures [22, 24] and dental attrition [25, 26]. The skeletal pathological phenomena were diagnosed according to the study of Ortner, Roberts and Manchester, as well as Waldron [27–29]. Skull morphological features were measured and described [22]. Stature estimations were made according to Shao and Zhang [22, 30].

### Isotope analysis

We selected both limb bone and ribs for each individual in M56 to conduct carbon and nitrogen stable isotope analysis (Table 1). The extraction of bone collagen was following Ambrose’s method [31]. The carbon and nitrogen content and the isotope ratios in the bone collagen were tested in the Key Laboratory of Western China’s Environmental System, Ministry of Education, Lanzhou University, with the Elemental Analyzer (vario EL cube) and Thermo Finnigan Flash DELTA plus XL mass spectrometer (Finnigan, Germany). Sulfanilamide was used as the reference material in the content measurement of C and N element. The C and N stable isotope ratio were calibrated by the international reference material IAEA-600, IAEA-cH-6 and IAEA-600, IAEA-N-2 respectively and a laboratory-made bone collagen standard sample (δ^13^C value –14.7±0.1‰, δ^15^N value 7.0‰±0.1‰) was inserted into every 10 samples tested, with the analytical errors all below ±0.2‰.

### Ancient DNA analysis

#### DNA extraction, NGS library preparation and enrichment

Sample preparation, DNA extraction and library preparation were carried out in the dedicated clean facilities specially designed for ancient DNA at Jilin University, following the standard procedure [32]. All samples were decontaminated by wiping the surfaces with 5% bleach before they were transferred to a dedicated clean room. DNA was extracted from teeth, following published protocol [33]. The Double-strand libraries were constructed according to the procedures described by Dabney et al. [34] and then sequenced on an Illumina HiSeq X Ten platform (Novogene, China) in the 150-bp paired-end sequencing design. IS5_reamp. P5 and IS6_reamp. P7 primers from Meyer and Kircher [35] were used. We enrich the mitochondrial genome using an in-solution [36] mtDNA capture kit (iGeneTech, China). For autosomal enrichment, we selected 70,000 SNPs from the 1240k panel [37]. The baits were designed and produced by iGeneTech company. The in-solution enrichment was conducted according to the manufacture’s protocol. The enriched samples were sequenced on an Illumina HiSeq X Ten platform (Novogene, China) in the 150-bp paired-end sequencing design.

#### NGS data processing

The raw sequencing reads were processed in EAGER v1.92.50 program [38]. Adapters were trimmed with AdapterRemoval v2.2.0 [39]. Reads shorter than 30bp were discarded. The trimmed data were then mapped to the human reference genome (GRCh37) using BWA 0.7.12 [40, 41]. Use DeDup v0.12.2 to remove duplicated reads [38]. Reads with a mapping quality higher than 30 were retained using SAMtools v 1.9 [41]. To minimize the bias caused by the deamination of ancient DNA, we trimmed the alignment reads according to the frequencies of C to T or G to A at both 5’ and 3’ ends to a degree that the damages at the end of the trimmed reads were identical to the baseline. Eight nucleotides were trimmed from both end of each sequence with bamUtils v1.0.13 [42]. We randomly called genotype for the SNPs in the 1240k panel [37, 43] from trimmed reads with high-quality bases (Q > 30) that implemented using pileupCaller (https://github.com/stschiff/sequenceTools).

#### Ancient DNA authentication

We identified the molecular damage which is typical of ancient DNA through mapDamage v2.0.6 [44] with default parameters. The contamination of mitochondrial sequences was assessed using schmutzi [45], an iterative likelihood-based method that jointed estimations of ancient DNA contamination and endogenous mitochondrial consensus sequences. We measured the nuclear genome contamination rate in male sample based on X chromosome data, as implemented in ANGSD v0.910 [46]. DNA molecules bearing deamination were selected using PMDtools (v.0.60) [47] with the “–threshold 3” parameter.

#### Genetic sexing and mitochondrial haplogroups analysis

We assessed the genetic sex of our samples by evaluating the ratios of reads aligned to the X chromosome compared to the total number of reads aligning to the autosomes (Rx ratio) [36].

For mtDNA haplogroup assignment, we utilized the Geneious Prime v11.1.3 with the “threshold: highest quality (60%)” parameter to generate mtDNA consensus sequences (https://www.geneious.com/) and then assigned mitogenome haplogroups with HaploGrep2 [48]. We checked the SNPs through IGV software [49] based on PhyloTree Build 17 [50].

#### Population genetic affinity analysis

The samples were first merged with published ancient genome-wide data for the 1240k panel [37, 43]. The merged dataset was then compared to modern populations in the Affymetrix Human Origins public dataset (593,124 autosomal SNPs) [51]. The principal component analysis was carried out using the “lsqproject” options in the smartpca program in Eigensoft v6.1.4 package [52]. We projected our ancient samples onto the variation of present-day Eurasians from published Human Origin dataset. The ADMIXTURE analysis [53] was performed on the ‘HumanOrigins’ dataset after pruning for linkage disequilibrium in PLINK [54] with parameters—indep-pairwise 200 25 0.4 which retained 304,935 SNPs. We ran ADMIXTURE with default 5-fold cross-validation (—cv = 5) and the number of ancestral populations ranged between K = 2 and K = 20 in 100 bootstraps. The outgroup f3-statistics were calculated using the qp3Pop (v435) programs in the ADMIXTOOLS v5.1 package using default parameters [55].

#### Genetic relatedness estimation

We estimated the genetic relatedness between the Shuangzhao individuals using pairwise mismatch rate analysis (PMR) [56]. The PMR approach estimates kinship by calculating the pairwise mismatch rate of haploid genotypes across autosomal SNPs. For each pair of individuals, we defined the PMR value by dividing the number of SNP sites for which two individuals have different alleles sampled by the total number of sites covered in both individuals. In general, the PMR value of the identical individuals (relatedness coefficient r = 1) should be half of that between the unrelated individuals (relatedness coefficient r = 0, identified as the population baseline, no inbreeding). Likewise, the PMR value for first- (relatedness coefficient r = 0.5) and second-degree relatives (relatedness coefficient r = 0.25) should be 3/4 and 7/8 of the baseline.

## Results

### Archaeological analysis on M56

#### The age determination of M56

Carbon-14 dating analysis conducted on the bone of R3 in M56 showed that the date of the remains was 1268–1072 cal BP (682-878AD, 2σ, Lab code: LZU20263), which could trace M56 back to the Gaozong to Xizong Period in the Tang Dynasty. This result is consistent with the archaeological findings: the plane structure of M56 is like a straight-back knife, with the east wall of the tomb passage and chamber on the same plane. This type of burial was common in the middle and late Tang Dynasty (8th century to early 10th century) [17]. In addition, the pagoda-shaped jar with a lid unearthed from M56 (Fig 1D) is different from that of the late Tang in terms of shape and craftsmanship. Integrating all pieces of evidence above, M56 can be dated to the Mid-Tang period (Dezong to Wenzong Period).

#### The social status of the individuals buried in M56

In the middle and late Tang Dynasty, rectangular earthen cave tombs were usually used for the burial of lower officials and common civilians [15]. The structure of M56 is simple, with a 2.06m long, 1–1.37m wide, and less than 9m^2^ tomb chamber. Only one rough pagoda-shaped jar was buried with hosts as grave goods. Inferring from the type of the burial and the quantity and traits of the funeral object, the social status of the individuals buried in M56 was identified as common civilians [15].

#### Osteobiographical analysis on the three human remains in M56

M56 contained the skeletal remains of three individuals, R1, R2, and R3, as shown in Fig 1B and 1C. R1 was an adult male, with an age at death of ~40–45 years old. The body was laid to its right side slightly. His cranium was on the right side of the spine and was shifted a little from the primary anatomical position (S1 Fig in S1 File). His right arm was pressed under his right ribs and spine. Since the male’s skeleton remained intact and articulated, it is considered a primary burial for R1. R2 was an adult female aged ~20–25 years old, lying on the left side of R1 on a lower layer. The body was not in the primary anatomical position, and some of the vertebrae, ribs, limb bones, as well as clavicles, were missing (S2 Fig in S1 File), which was caused by disturbance or secondary burial. After the exhumation of R1, the remains of R3 lying below R1 were exposed (S1 Fig in S1 File). R3 was an adult female with an age at death of ~40–45 years old. Her limb bones were roughly in the primary anatomical position, but other bones were in disorder, for example, her mandible was near the feet. The same as R2, some bones of R3 were also absent, such as partial vertebrae, ribs, and fibulae (S2 Fig in S1 File). She may also have experienced disturbance or secondary burial. The three skeletons were not situated at the base of the tomb, but were stacked on the sediment resulting from the collapse of the soil layer at the apex of the tomb. They were located in a corner of the chamber (S1A Fig in S1 File). This implied that a process of water inflow occurred in the tomb, causing human bones drift and superposition. R2 and R3 may have originally been positioned on either side of R1. According to taphonomic information, M56 was a primary burial site with no indication of secondary use. Under the peaceful social conditions in this region of the Tang Dynasty, it is highly improbable that the three individuals died simultaneously. Considering the disturbed conditions of R2 and R3, as well as the absence of certain bones, it is speculated that they were relocated from their original burial site to M56, together with R1. After being buried in M56, they experienced another displacement due to the collapse of the top of the tomb and water invasion. Referring to R1’s situation, this displacement did not have a significant impact on the relative position of individual bones.

Osteometric analysis is important for comprehending the physical traits of both individuals and populations. Due to the role of genes and environment, groups exhibiting similar craniofacial morphology may have closer relationship compared to groups that exhibit dissimilar morphology [57]. The physical features of R1 can be summarized as a moderate cranial length (mesocrany, index:78.75), a high cranium (hypsicranial, index:76.40), and a moderate cranial width (metriocrany, index:97.01). These features were typical of the ancient inhabitants of the Central Plains and Northern China [58]. The craniofacial morphology characters of the two female skulls are very similar, in being dolichocrany, hypsicranial and narrow cranial type (dolichocrany, index:74.93/72.76; hypsicrany, index:77.58/78.30; acrocrany, index:103.53/107.61). These two skulls have a higher and relatively flat degree faces (orthognathous, index:87.90/87.00). The craniometry and appearance of skulls indicates an east Asian affinity (S1 Table in S1 File). To further explore their possible origin, the Euclidean distance analysis based on the craniometric data of the female skulls from the Shuangzhao and other historic groups of China was carried out. The results showed that the Shuangzhao group had closest distance with the Central Plains group (Zhengzhou) of the Tang Dynasty (S2 Table in S1 File). Statures of R1, R2 and R3 were estimated as 165.04 cm, 158.61 cm and 162.09 cm, respectively. Compared to the other Chinese historic groups, the Shuangzhao group had relatively low sexual dimorphism of stature (S3 Table in S1 File).

**Fig 2:**
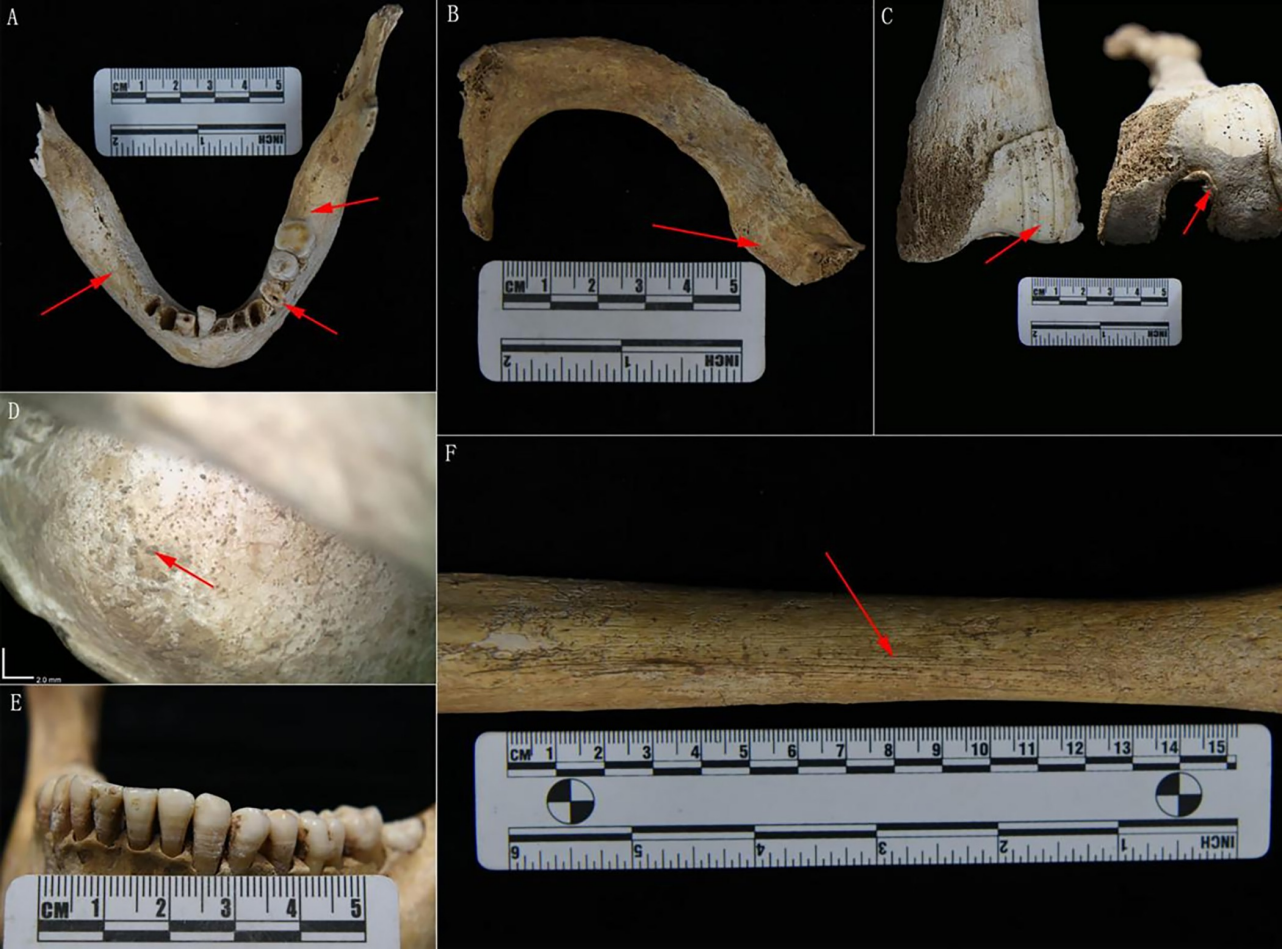
Pathological lesions on the bones and teeth of individuals in M56. A. Extensive attrition on the left premolar of the mandible of R3, accompanied with antemortem teeth loss, and part of the sockets have been completely healed. B. Ossification of the left first rib cartilage of R3. C. Obvious osteoarthritis in the left femur of R1. D. Cribra orbitalia of R2 with most of the holes have smooth margins and nearly closed. E. Enamel hypoplasia on the labial surface of the mandibular teeth of R2. F. Periostitis on the left tibia of the R2.

Owing to the relatively elderly age at death, some signs of diseases associated with senectitude were observed on the skeletons of R1 and R3, for instance, teeth loss, excessive wear of teeth (Fig 2A), as well as ossification of rib and thyroid cartilage (Fig 2B). Eburnation was prominently observed on the patellar surface of the femora in R1, along with osteophytes surrounding the patella and apparent bone resorption on the articular surface, leading to the preliminary diagnosis of osteoarthritis (Fig 2C). Small orifices characterized by smooth margins and almost complete closure were discovered on both R2 and R3 orbits (Fig 2D, S3 Fig in S1 File), which could possibly indicate the presence of cribra orbitalia in its healing phase. Additionally, the labial surface of the mandibular incisors and canines were observed to have transverse lines and grooves, which serve as indicators of enamel hypoplasia (Fig 2E). Signs of periostitis were observed on the tibiae of R1 and R2, and the presence of lamellar bone indicated a chronic and remodeled condition (Fig 2F). From the perspective of bioarchaeology, a variety of factors may lead to the formation of enamel hypoplasias, cribra orbitalia, and periostosis, which are commonly recognized as indicators of enduring surviving stress [59].

### Stable isotope analysis

Isotopic analysis was performed on ribs and limb bones. These samples were successfully used for collagen extraction. The results of isotope analysis showed that the δ^15^N values of the three individuals ranged between 8.6‰ and 8.8‰ (average 8.8‰±0.1‰), suggesting a relatively low consumption of animal protein (Table 1, S4 Table in S1 File). The δ^15^N values among the three individuals were very similar, and no sex-based differences were observed. The mean δ^13^C value from the ribs was -15.0‰±0.81‰, and mean value from the limbs was -15.3‰±1.42‰, which indicated a consumption of both C_3_ and C_4_ plants (Table 1). According to the historic records, in the Tang Dynasty, wheat and millet were the main crops planted in Xianyang, and rice was also planted there [60]. The δ^13^C and *δ*^15^N data from ribs reflect the dietary information of the 2 to 5 years before death, while those from limbs reflect the condition for about 10 years before death [61, 62]. The δ^13^C and *δ*^15^N values from ribs and limbs of these three individuals varied slightly.

**Table 1:** Carbon and nitrogen stable isotope values from the individuals in M56.

Individual	Sex	Age at death (years)	Skeletal element	δ^13^C	δ^15^N	N%	C%	C/N
R1	M	40–45	rib	-15.7	8.9	16.1	43.9	3.2
			metacarpal	-16.6	8.8	15.9	43.9	3.2
R2	F	20–25	rib	-14.1	8.8	16.3	44.1	3.2
			fibula	-13.8	8.6	15.9	43.3	3.2
R3	F	40–45	rib	-15.1	8.8	15.6	42.8	3.2
			metacarpal	-15.6	8.8	16.5	44.5	3.2

### Ancient DNA analysis on the three individuals in M56

#### Ancient DNA authentication and contamination assessment

Three individuals excavated from M56 were shotgun sequenced. To authenticate the sequenced fragments, the terminal substitution rate was calculated. A typical double-stranded aDNA library pattern of excess cytosine to thymine misincorporation at the 5’ end, and guanine to adenine misincorporation at the 3’ end was observed, which demonstrated the characteristics of ancient DNA (S3-S5 Figs in S1 File). Furthermore, the average length of the obtained DNA was 65–76 bp, which was consistent with the general characteristics of the average length of the ancient DNA. The degree of mitochondrial DNA contamination was estimated ranging from 1% to 2.5%. The X-chromosomal contamination test for the male individual was not informative because of the limited number of X-chromosomal SNPs covered by at least two sequences.

#### Sex determination and uniparental genetic analyses

To investigate the genetic profile of the Tang Dynasty inhabitants, we retrieved the whole genomic sequences of the samples to an average coverage from 0.005X to 0.03X (Table 2). We conducted a biological sex determination by evaluating the Rx ratio. As a consequence, R1 was assigned as male (Rx: 0.506), while R2 and R3 were assigned as female (Rx: 0.995, Rx: 0.926, respectively), which confirmed the results of anthropological sex identifications.

**Table 2:** Details of the genomic data generated in this study.

Sample ID	Total reads	Genetic sex	Endogenous (%)	Mean coverage	1240k SNPs	70k SNPs	mtDNA coverage	mtDNA haplogroup	mtDNA Contamination
M56-R1	97473780	Male	3.64	0.016	78583	52333	468	G1a1	0.025
M56-R2	57529698	Female	17.42	0.031	113648	65400	1508	D4a2e	0.01
M56-R3	100087374	Female	2.58	0.005	51770	40360	193	M9a	0.019

We first reconstructed the mitochondrial genome by in-solution enrichment to explore the matrilineal relatedness of these individuals. We obtained a nearly complete mitochondrial genome (96.9%-99.6%) with the average coverage ranging from 193 × to 1508 × (Table 2). These mtDNA genome sequences were not identical and belonged to haplogroup G1a1, D4a1e and M9a, respectively. Haplogroup D4 is prevalent in the north of East Asia and has a relatively higher frequency in the Neolithic Longshan and Yangshao populations [63–65]. M9a is distributed widely in mainland eastern Asia, Japan, Tibet, and surrounding regions [64–66]. G1a1 has been detected in Siberia and East Asia [67, 68]. In addition, both haplogroup G1a1 and M9a were found among the Yangshao population [63, 69].

#### Autosomal genetic analyses

**Fig 3:**
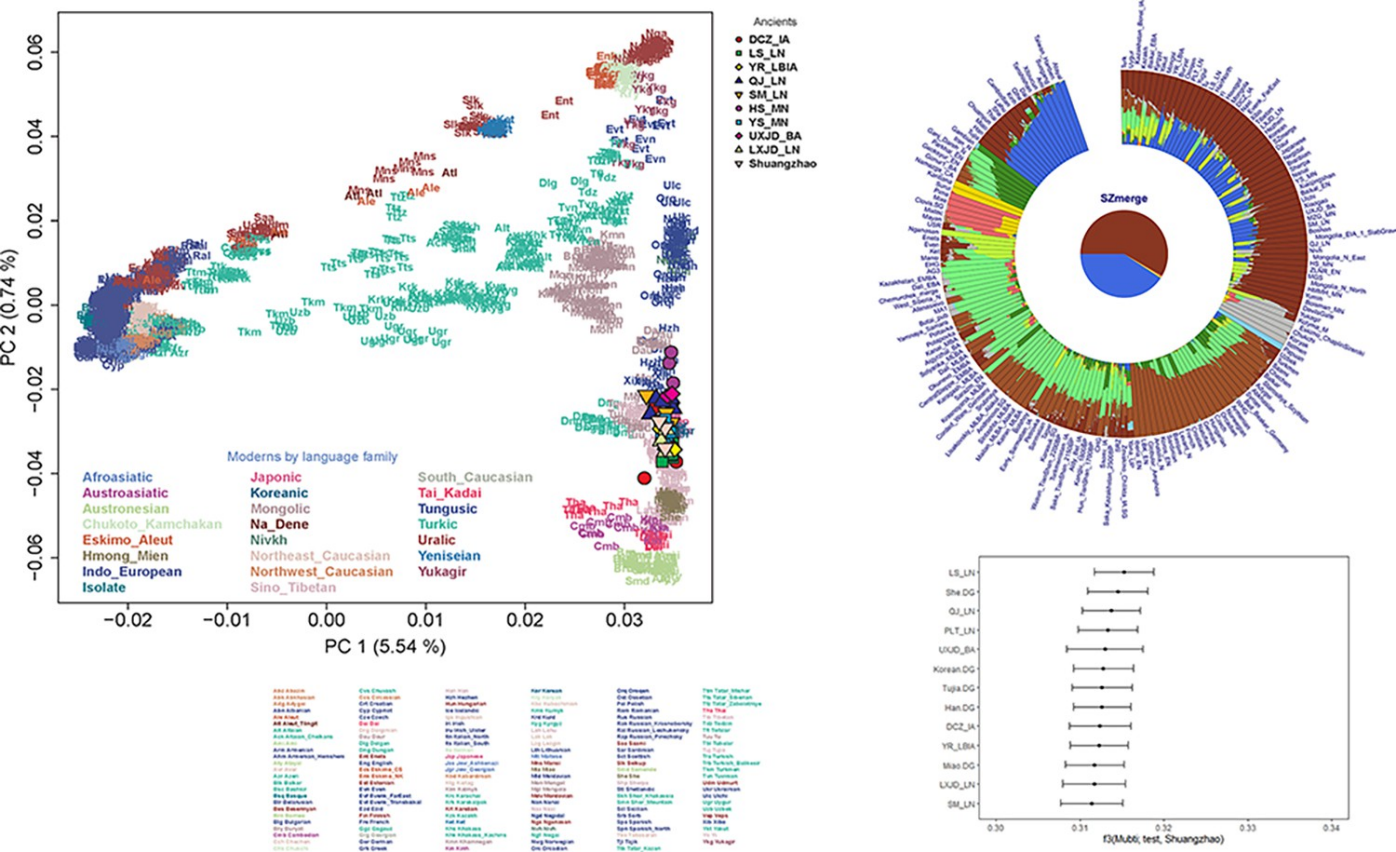
Genetic structure of the Shuangzhao individuals. A. The PCA was constructed from present-day Eurasians in the Human Origins dataset, and the ancient individuals were projected onto the top PCs. B. Plot of ADMIXTURE (K = 10, with minimum CV error) results (S5 Table in S1 File). C. Shared Genetic drift between the Shuangzhao individuals and worldwide representative populations.

We carried out principal component analysis (PCA) to assess the genetic relationship between the Shuangzhao population and other Eurasian groups (S7 Table in S1 File) by projecting them onto present-day Eurasian variation. The Shuangzhao individuals fell within the variations of eastern Eurasians and clustered with the Neolithic Central Plains populations, such as the Longshan and Yangshao populations (Fig 3A). We then performed ADMIXTURE analysis to get a detailed overview of the ancestry composition. We observed the lowest CV error at K = 10. Consistent with the PCA, the Shuangzhao individuals showed a genetic profile that was similar to the ancient Central Plains populations (Fig 2B). We applied outgroup *f3*-statistics in the form of *f3*(Mbuti; test, Shuangzhao) to further explore the genetic relationship between the shuangzhao and Eurasian populations. The result of *f3*-statistics indicated that the Shuangzhao individuals had a close relationship with northern East Asians, and shared the closest affinity with the Longshan population (Fig 3C). These observations were also supported by the ADMIXTURE analysis and symmetric *f4*-statistics (Fig 3B, S8 Table in S1 File). We also compared the genetic relationship between the Shuangzhao and modern Chinese populations. The symmetric *f4*-statistics in the form *f4*(Mbuti, X; Han, Shuangzhao) showed that compared to the Shuangzhao population, modern Han had a significant genetic affinity with populations from south China and South East Asia, such as Ami, Atayal, and Dai, etc. (S8 Table in S1 File). This genetic pattern was also observed in the PCA and ADMIXTURE analysis.

Based on the genomic data, we’ve found that the three individuals in M56 all fall into the East Asian gene pool, and are clustered with other ancient Central Plains populations such as Longshan individuals and modern Chinese populations such as Han, Naxi, Lahu, Yi, Tibetan and Tujia, all belong to Sino-Tibetan speakers. There is no sign of the exotic genetic components from outside the Central Plains.

#### Genetic relatedness estimation

The three individuals (one male and two females) sampled from the Shuangzhao cemetery were buried in the same tomb. Exploring the potential relationship of these individuals was vital for understanding the funeral rites of the Tang Dynasty.

We tried to use autosomal data to estimate genetic relationships among the three individuals. Due to the relatively low endogenous DNA content, an in-solution DNA capture was carried out using a 70k SNPs enrichment kit. We successfully recovered 52,333, 65,400, and 40,360 SNPs from R1, R2 and R3, respectively. Meanwhile, we also genotyped these 70k SNPs from the published genomic sequences of genetically related and unrelated ancient samples from the Central Plains to generate a baseline (S6 Table in S1 File). To minimize the artificial bias due to the high missing rate of ancient DNA, we restricted our analysis to individual pairs with at least 10,000 overlapping SNPs. We deduced the kinship of the Shuangzhao individuals through Pairwise mismatch rate (PMR). The PMR values of the Shuangzhao pairs range from 0.3018 to 0.3035 (Table 3), which were approximate with the baseline of unrelated ones (Table 3, S7 Fig in S1 File). This result further proved that the Shuangzhao individuals share no relatedness to each other, albeit buried in the same grave.

**Table 3:** Pairwise mismatch rate (PMR) among the Shuangzhao individuals.

Sample ID	Sample ID	nSNPs	nmismatch	pmismatch
M56-R1	M56-R2	49071	14891	0.30346
M56-R1	M56-R3	30539	9241	0.3026
M56-R2	M56-R3	37860	11429	0.30188

## Discussion

Based on the solid framework established by rigorous field excavation, sorting and research, the collaboration across disciplines can provide a more comprehensive understanding of cultural diffusion, social organization, and even cultural practices, including family burial customs, and marriage practices, etc. [70]. The Shuangzhao cemetery is located in Xianyang, which was an important transportation junction connecting the famous Silk Road and other important post roads in the Tang Dynasty. Therefore, as an international metropolis, Xianyang had a very diverse population [3]. In addition to local civilians and government officials, there were also foreign envoys, businessmen and craftsmen who had come to live here [3]. Therefore it was important to understand the social identity of the Shuangzhao individuals of M56. The burial objects and the form of the tomb, which is commonly used to identify social status in the Tang Dynasty, revealed that the Shuangzhao cemetery was used by local civilians rather than nobles or high-status people in the society. The analysis of the genomic profile of these individuals showed that the individuals buried in the tomb had typical genetic characteristics of the Central Plains, and there was no kinship among these three co-buried individuals. In addition, the isotopic results indicated that the diets of the three individuals were similar, mainly based on C_3_ and C_4_ plants with relatively low animal protein. Combining these pieces of evidence, we confirm that the people buried in M56 were common civilians of the Tang Empire.

Joint burial was a common form of funeral in the Tang Dynasty [18, 19]. The most common pattern of joint burial is that husband and wife are buried together [18–20]. Other family members such as parents and children, siblings, mothers-in-law and daughters-in-law were also found buried in a single grave [18]. In M56, relationship between the one male (R1) and two females (R2 and R3) buried together is of great interest. Genomic analysis showed no genetic association between the three individuals. Furthermore, no sacrificial slaves were permitted into the tombs of civilians in the Tang Dynasty [19]. According to the Marriage Law of the Tang Dynasty [71], a man could only have one legal wife. Remarriage was allowed only after the death of the first wife, and the family status of the second wife should be the same as that of the first wife [71]. It was permitted that men of Tang Dynasty took concubines, whose status was inferior to that of his wife and they were not allowed to be buried with the couple. From these clues, we infer that M56 is a joint burial for a husband with his first and second wives. Based on the location, age and isotope data of all individuals, we can deduce the burial process of M56: R2 should be the man’s first wife. Unfortunately, she died at a very young age (approximately 25 years old) and was buried in a temporary place. After her husband died, her descendants transferred her remains back into the joint burial. It is consistent with the custom of returning the remains in the Tang Dynasty [20, 72]. The second wife R3 also died before the man, and was reburied in the joint burial after the death of the husband. The husband R1 was buried in the tomb directly after his death.

This case of multi-burial allows us to explore the funeral customs of the Tang Empire. The two wives reburied following their husband’s death reflected a patrilocal social organization and the dominant position of the husband in a family in the Tang Dynasty. On the other hand, there were no distinctive differences in the grave goods, funeral rites and the diet structure among the husband and his wives. This may be associated with the socio-political economy and the diversity of population sources of the Tang society [3], which led to the fact that the status of women in the Tang Empire was relatively higher than that of other dynasties in Chinese history.

## Conclusion

In summary, we have employed a multidisciplinary approach to investigate the Shuangzhao cemetery and provided sociocultural insights into the Tang Empire, one of the most splendid empires in Chinese history. Based on the integrated lines of evidence, we were able to infer the social status and the relationships of the co-buried individuals, and explore the burial customs and social organization of the Tang Dynasty. In this case, we restored a scenario of civilian life in historic China. We believe that more samples from different sites should be instrumental for researchers to obtain further and more precise information about Tang Empire.

## Supporting information

S1 File (ZIP)
