# Supplementary material for: A multidisciplinary study on the social customs of the Tang Empire in the Medieval Ages
Source: PLoS One. 2023 Jul 26;18(7):e0288128. doi: 10.1371/journal.pone.0288128 (PMC10370703; doi:10.1371/journal.pone.0288128)
Supplement: S1 File — (ZIP) [file pone.0288128.s001.zip › Supporting Information/S2_Fig.pdf.pdf]

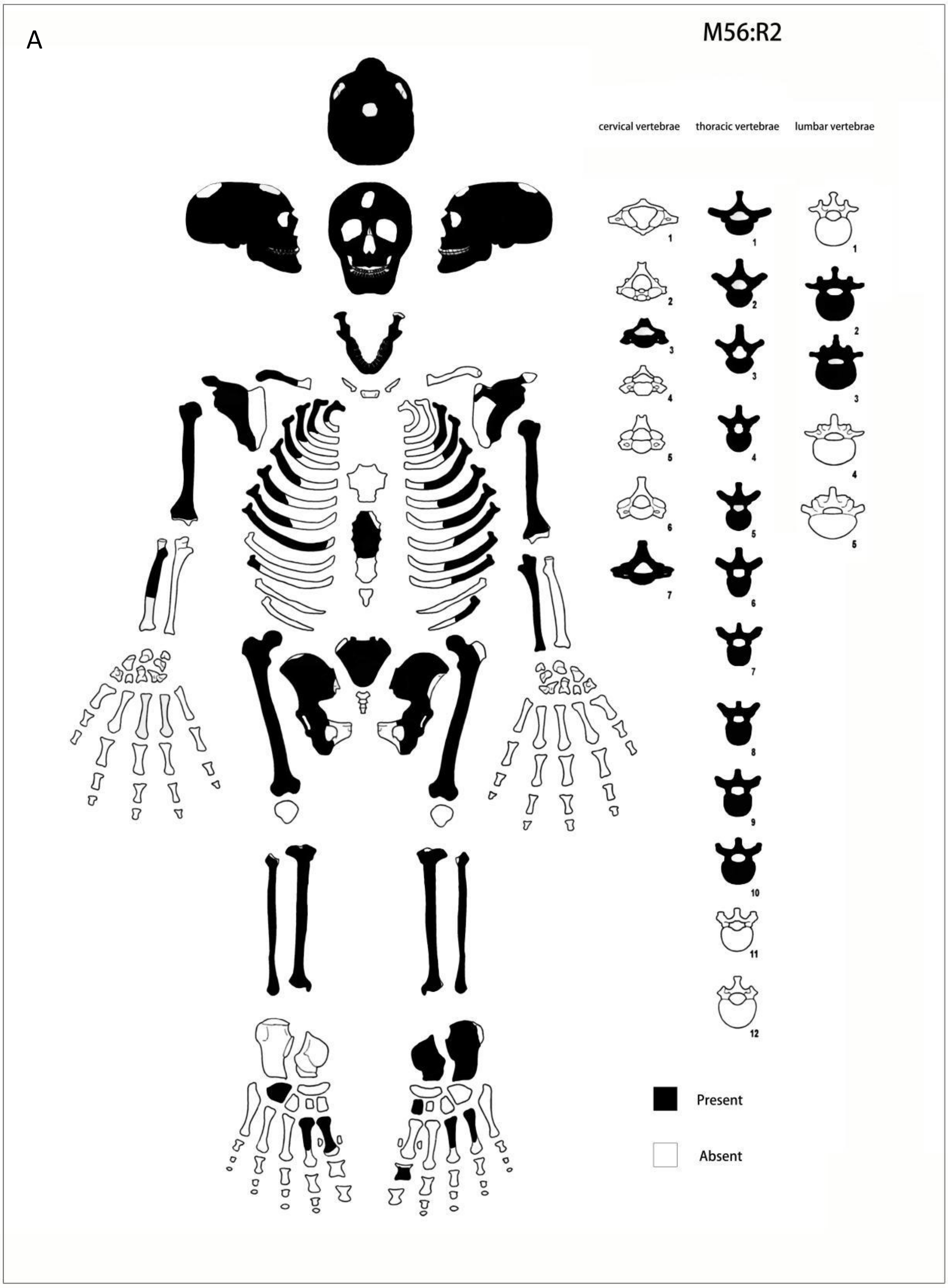
A
M56:R2
cervical vertebrae
thoracic vertebrae
lumbar vertebrae
Present
Absent

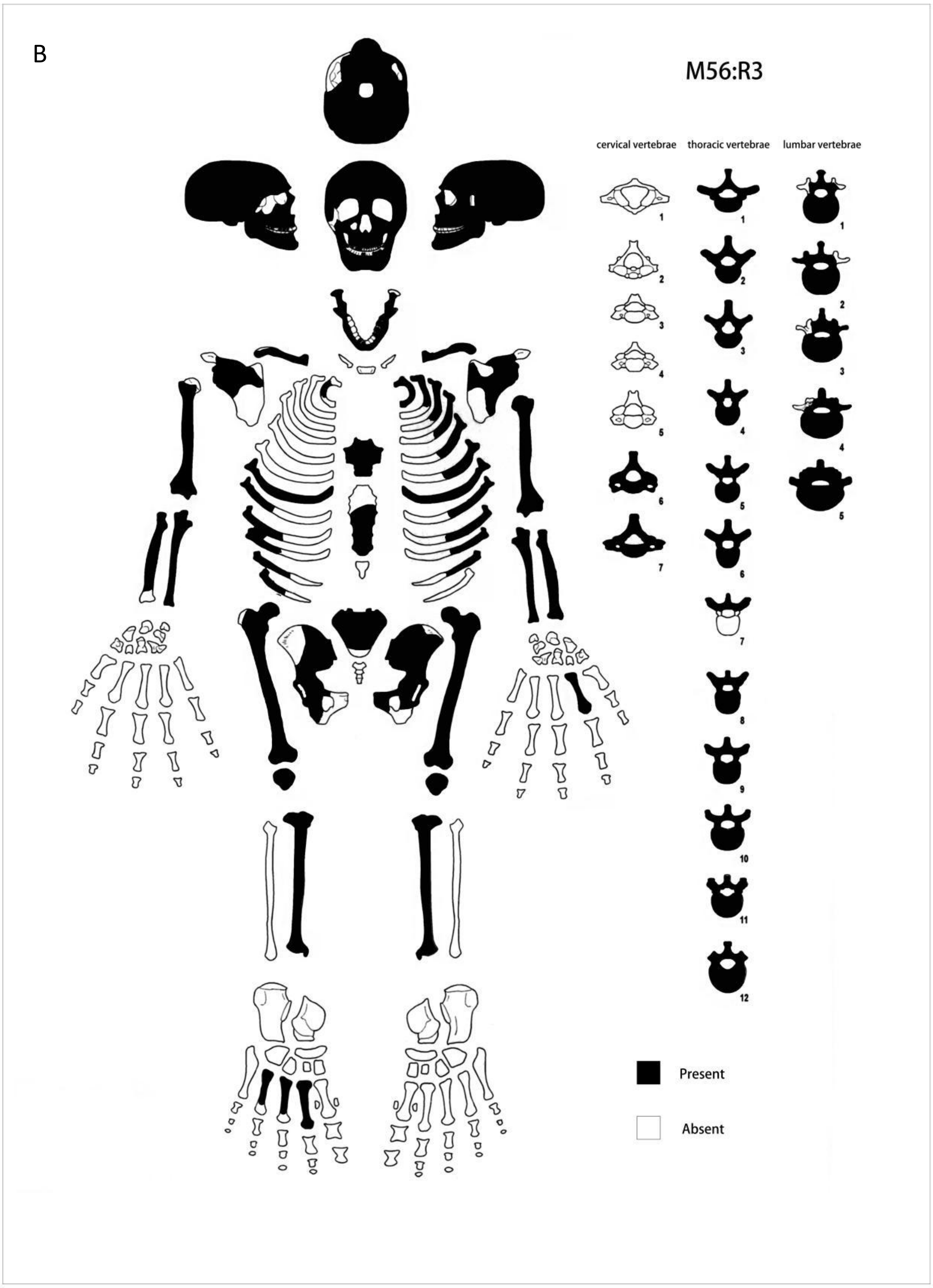

Skeleton line drawing from the file of URA 376 CNRS,according to T.S.Constandse-Westermann and C.Meikeljohn,modified from M.Guillon,P.Sellier and P.Courtaud,informatization M.Coutureau,AFAN
