# Supplementary figures and images for: A multidisciplinary study on the social customs of the Tang Empire in the Medieval Ages
Source: PLoS One. 2023 Jul 26;18(7):e0288128. doi: 10.1371/journal.pone.0288128 (PMC10370703; doi:10.1371/journal.pone.0288128)

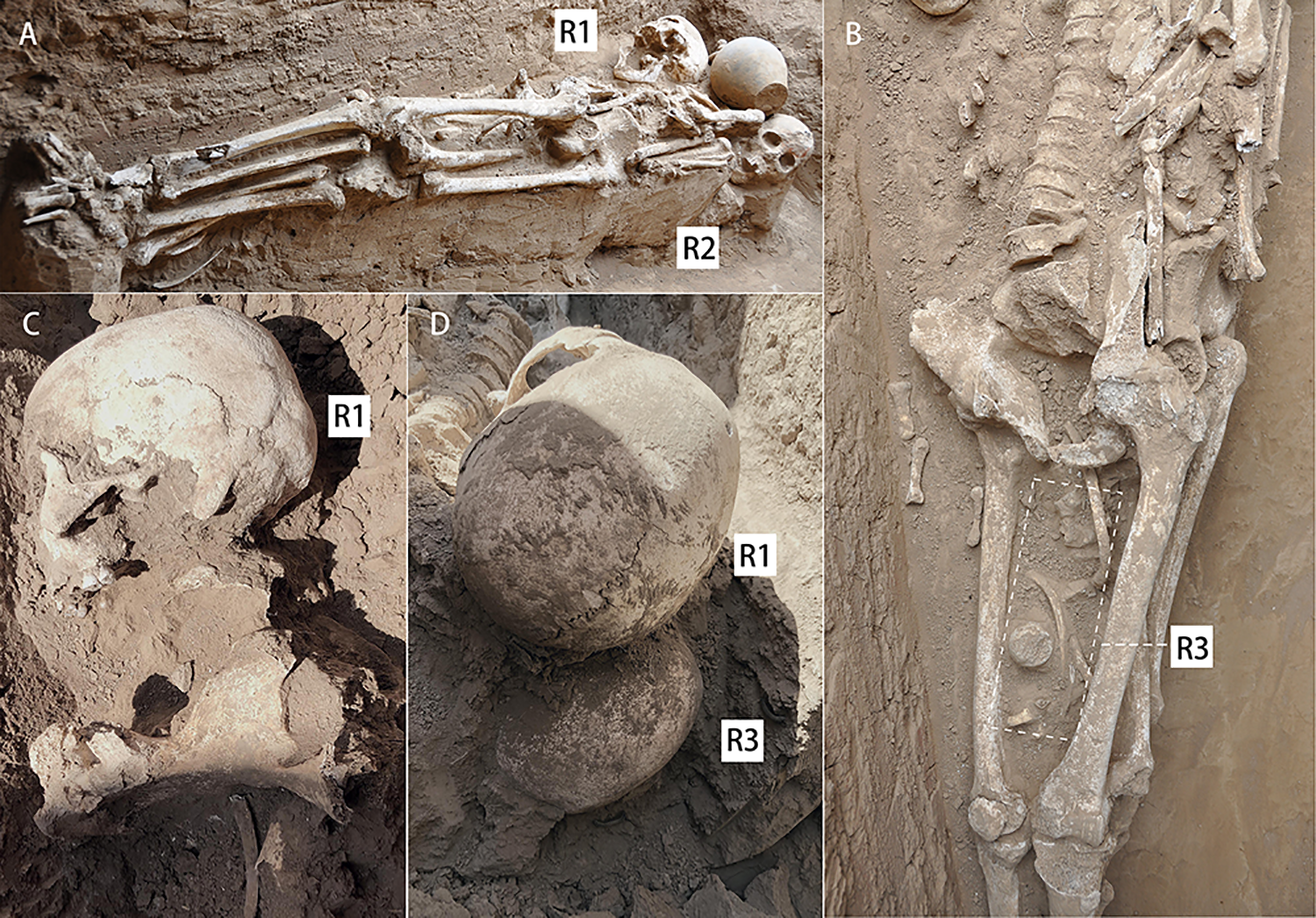

Supplement: S1 File — (ZIP) [file pone.0288128.s001.zip › Supporting Information/S1_Fig.tif.tif]

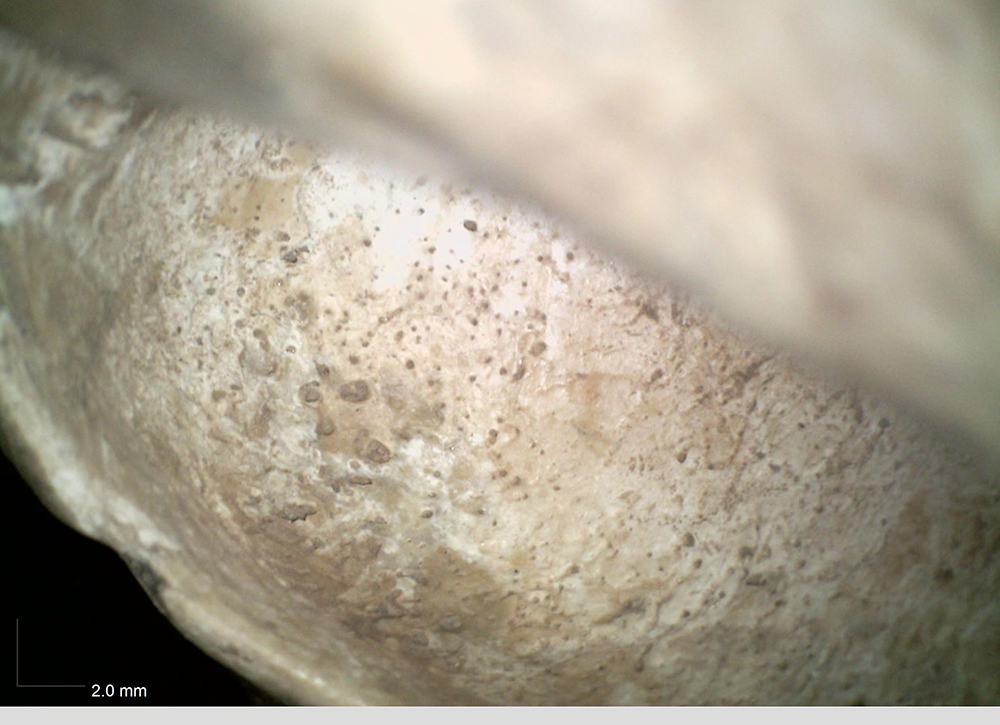

Supplement: S1 File — (ZIP) [file pone.0288128.s001.zip › Supporting Information/S3_Fig.tif.tif]

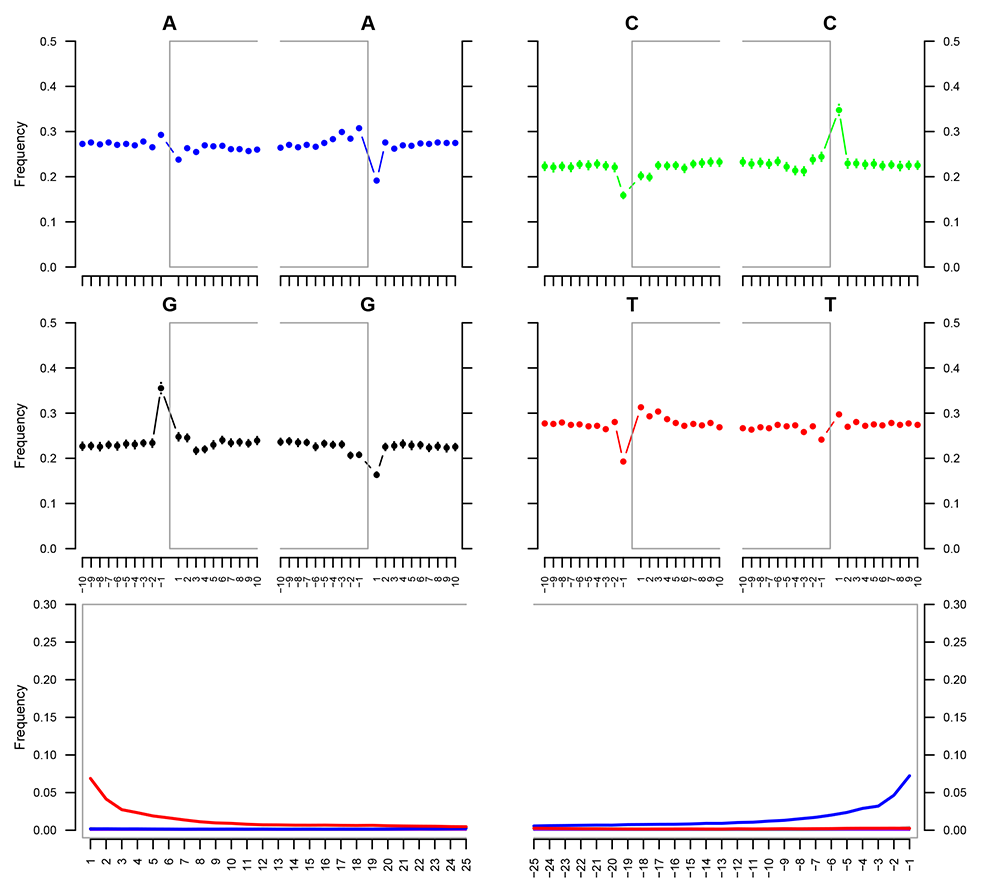

Supplement: S1 File — (ZIP) [file pone.0288128.s001.zip › Supporting Information/S4_Fig.tif.tif]

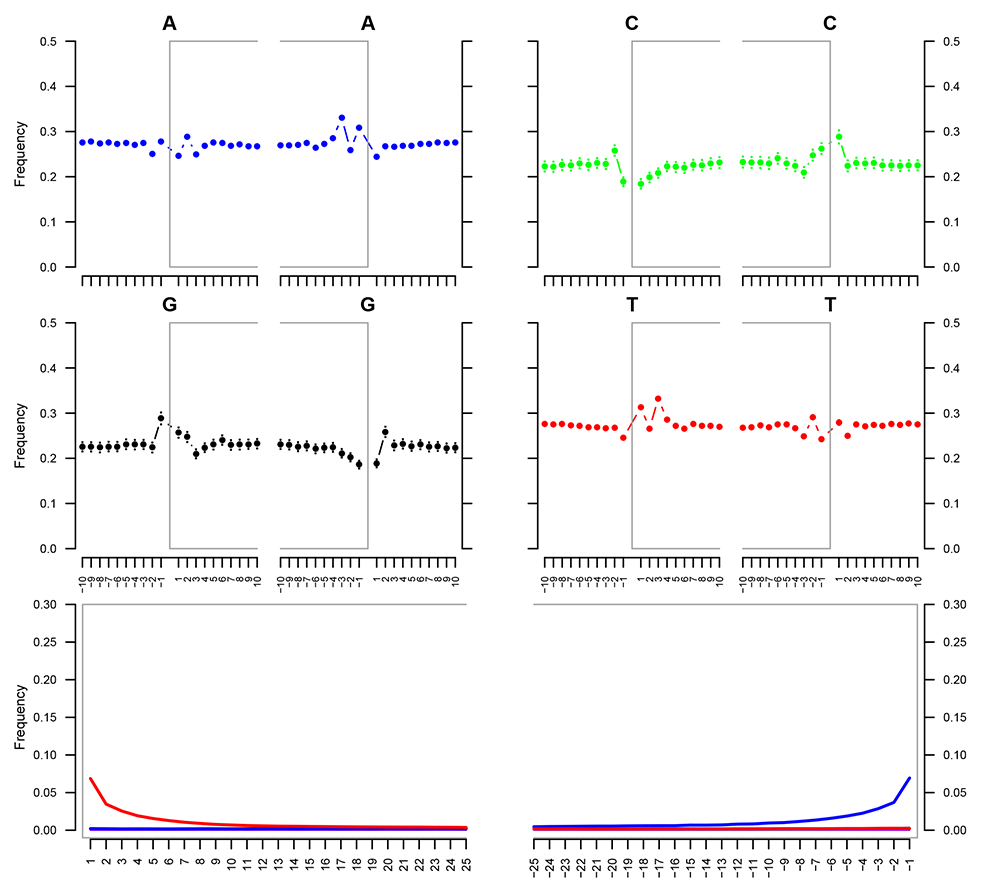

Supplement: S1 File — (ZIP) [file pone.0288128.s001.zip › Supporting Information/S5_Fig.tif.tif]

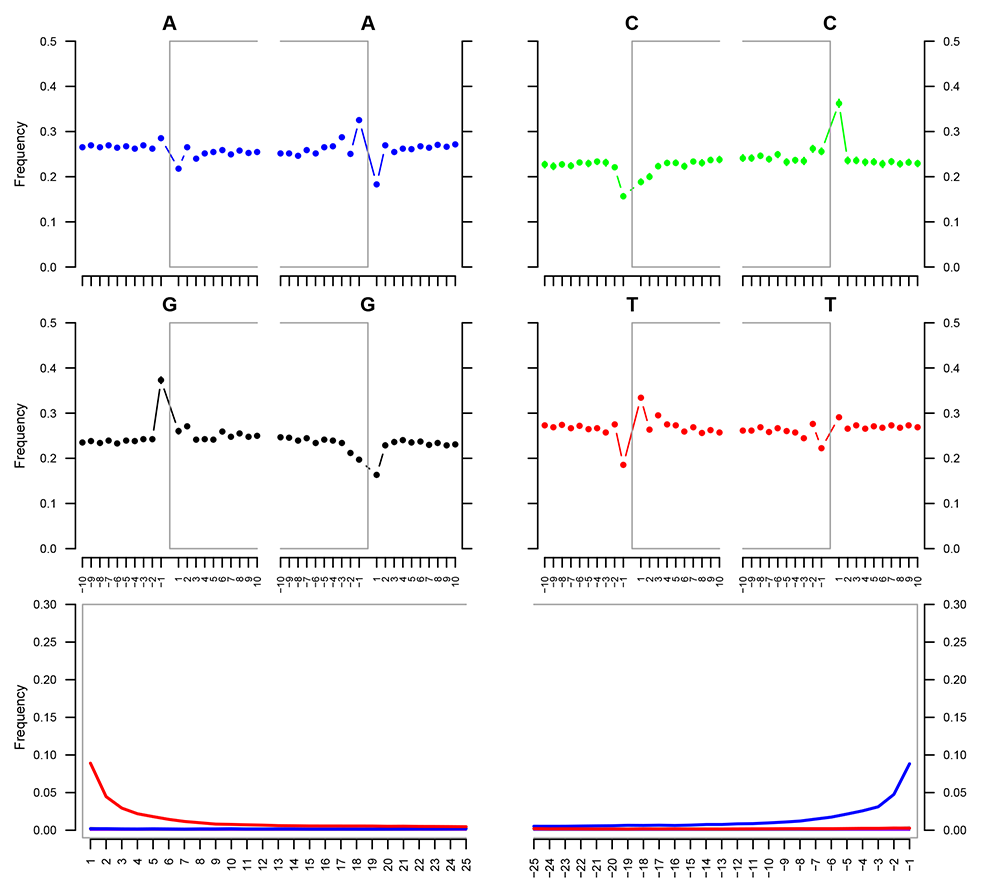

Supplement: S1 File — (ZIP) [file pone.0288128.s001.zip › Supporting Information/S6_Fig.tif.tif]

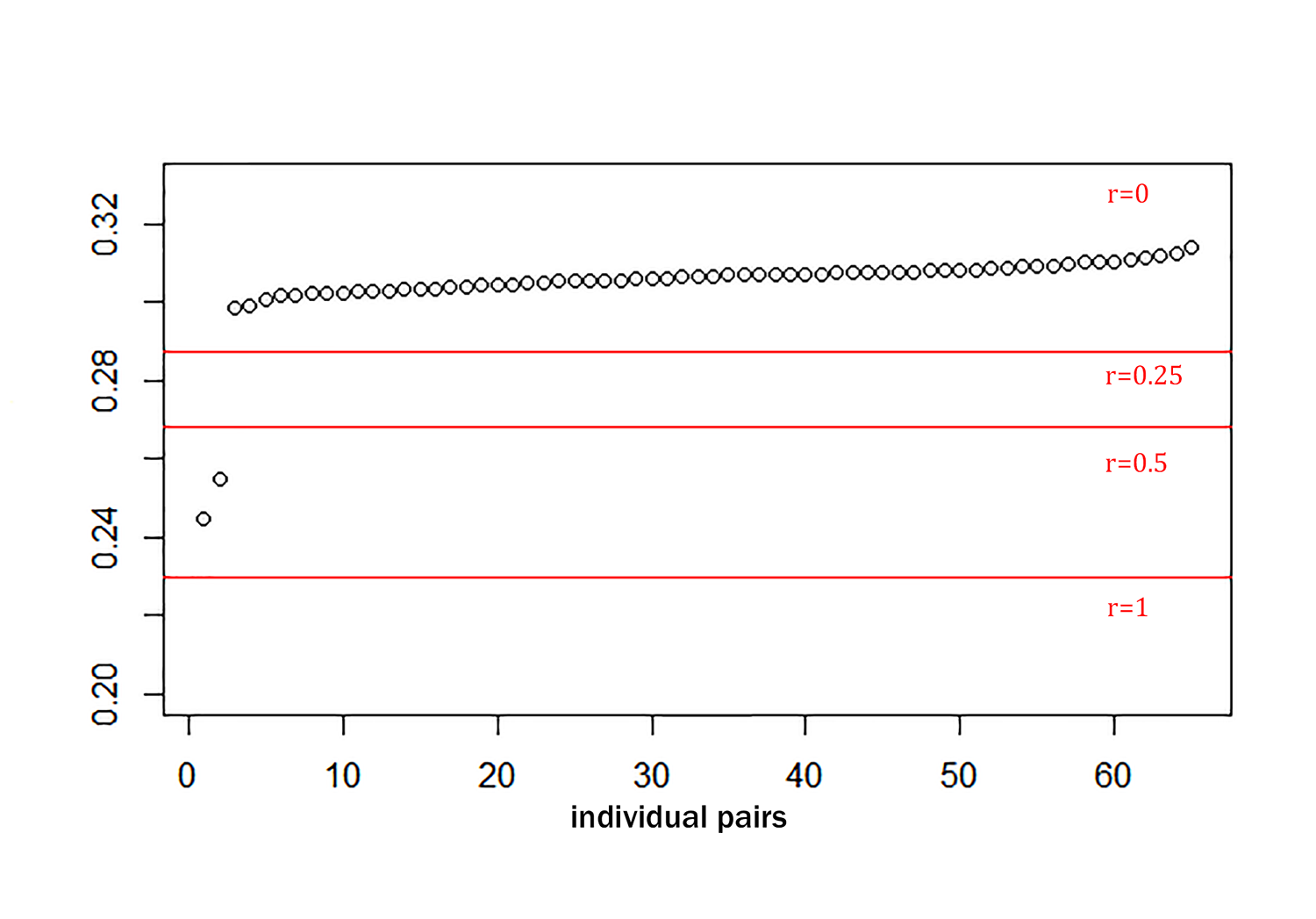

Supplement: S1 File — (ZIP) [file pone.0288128.s001.zip › Supporting Information/S7_Fig.tif.tif]
